# Protection against Hypoxia-Reoxygenation Injury of Hippocampal Neurons by H_2_S via Promoting Phosphorylation of ROCK_2_ at Tyr722 in Rat Model

**DOI:** 10.3390/molecules27144567

**Published:** 2022-07-18

**Authors:** Meng Xue, Shuo Chen, Jiaojiao Xi, Qianjun Guan, Wei Chen, Yan Guo, Zhiwu Chen

**Affiliations:** Department of Pharmacology, School of Basic Medical Sciences, Anhui Medical University, Hefei 230032, China; xuemeng@stu.ahmu.edu.cn (M.X.); chenshuo@stu.ahmu.edu.cn (S.C.); 1945010136@stu.ahmu.edu.cn (J.X.); 2045010101@stu.ahmu.edu.cn (Q.G.); 2045010105@stu.ahmu.edu.cn (W.C.)

**Keywords:** H_2_S, protection, hypoxia-reoxygenation injury, neuron, phosphorylation, ROCK_2_ at Tyr722

## Abstract

The RhoA-ROCK signaling pathway is associated with the protective effects of hydrogen sulfide (H_2_S) against cerebral ischemia. H_2_S protects rat hippocampal neurons (RHNs) against hypoxia-reoxygenation (H/R) injury by promoting phosphorylation of RhoA at Ser188. However, effect of H_2_S on the phosphorylation of ROCK_2_-related sites is unclear. The present study was designed to investigate whether H_2_S can play a role in the phosphorylation of ROCK_2_ at Tyr722, and explore whether this role mediates the protective effect of H/R injury in RHNs. Prokaryotic recombinant plasmids ROCK_2_^wild^-pGEX-6P-1 and ROCK_2_^Y722F^-pGEX-6P-1 were constructed and transfected into *E. coli* in vitro, and the expressed protein, GST-ROCK_2_^wild^ and GST-ROCK_2_^Y722F^ were used for phosphorylation assay in vitro. Eukaryotic recombinant plasmids ROCK_2_^Y722^-pEGFP-N1 and ROCK_2_^Y722F^-pEGFP-N1 as well as empty plasmid were transfected into the RHNs. Western blot assay and whole-cell patch-clamp technique were used to detect phosphorylation of ROCK_2_ at Tyr722 and BK_Ca_ channel current in the RHNs, respectively. Cell viability, leakages of intracellular enzymes lactate dehydrogenase (LDH), and nerve-specific enolase (NSE) were measured. The H/R injury was indicated by decrease of cell viability and leakages of intracellular LDH and NSE. The results of Western blot have shown that NaHS, a H_2_S donor, significantly promoted phosphorylation of GST-ROCK_2_^wild^ at Tyr722, while no phosphorylation of GST-ROCK_2_^Y722F^ was detected. The phosphorylation of ROCK_2_^wild^ promoted by NaHS was also observed in RHNs. NaHS induced more potent effects on protection against H/R injury, phosphorylation of ROCK_2_ at Tyr722, inhibition of ROCK_2_ activity, as well as increase of the BK_Ca_ current in the ROCK_2_^Y722^-pEGFP-N1-transfected RHNs. Our results revealed that H_2_S protects the RHNs from H/R injury through promoting phosphorylation of ROCK_2_ at Tyr722 to inhibit ROCK_2_ activity and potentially by opening channel currents.

## 1. Introduction

Cerebral ischemia-reperfusion (I/R) injury is mainly caused by the restoration of blood perfusion following brain tissue ischemia [1]. The pathophysiological mechanism of cerebral I/R injury is extremely complex and includes the oxidative stress response, intracellular calcium overload, toxic effects of excitatory amino acids, apoptosis, mitochondrial dysfunction, inflammatory reaction, and so on [2,3]. Cerebral I/R injury can cause apoptosis of hippocampal nerve cells and affect nerve function [4]. Protecting nerve cells from H/R injury is an important new strategy for treating ischemic brain injury.

H_2_S is a gaseous signaling molecule found in mammals that is involved in the regulation of various physiological functions and pathological processes in the nervous system and cardiovascular system [5]. Endogenous H_2_S is catalyzed by cystathionine-β-synthase (CBS), cystathionine-γ-lyase (CSE), and 3-mercap topyruvate thitransferase (3-MST) [6]. Previous studies have shown that H_2_S protects endothelial cell from hypoxic injury by inhibiting the RhoA-ROCK signaling pathway [7]. Endogenous H_2_S can inhibit the RhoA-ROCK signaling pathway in cerebrovascular vascular smooth muscle, cells, and cause hyperpolarization and vasodilation [8,9]. Moreover, H_2_S can improve cerebral vasoconstriction and diastolic dysfunction in cerebral ischemic mice and play a protective role against cerebral ischemic injury [10].

The RhoA-ROCK signaling pathway composed of RhoA and ROCK is an important target in cells. ROCK has two subtypes, ROCK_1_ and ROCK_2_, among which ROCK_2_ is mainly distributed in the brain and blood vessels [11]. The ROCK inhibitor fasudil can reduce neuronal apoptosis and promote neuronal growth [12,13], suggesting that inhibition of the RhoA-ROCK signaling pathway can reduce nerve cell injury. Previous research indicated that H_2_S can inhibit the RhoA-ROCK signaling pathway and produce a protective effect against ischemic brain injury, but the detailed mechanism by which H_2_S inhibits the RhoA-ROCK signaling pathway is not clear [14].Phosphorylation of the RhoA protein at Ser188 can reduce its translocation from the cytoplasm to the cell membrane, and prevent its activation [15]. Our recent studies have shown that H_2_S can significantly promote phosphorylation of the RhoA protein at Ser188 and inhibit RhoA activation [16].

Tyrosine phosphorylation at tyrosine residue 722 (Tyr722 or Y722) of ROCK_2_ protein can reduce the activation of ROCK_2_ [17]. However, it is not clear whether H_2_S can affect tyrosine phosphorylation at Tyr722 of ROCK_2_ protein to regulate the ROCK_2_ activity. Therefore, the present study was designed to investigate the ROCK_2_ inhibition mechanism of H_2_S protecting RHNs from H/R injury, especially focusing on the effect of H_2_S on phosphorylation of ROCK_2_ at Tyr722.

## 2. Results

### 2.1. Expressions of GST-ROCK_2_^wild^ and GST-ROCK_2_^Y722F^ in E. coli and Effects of NaHS on Their Phosphorylation In Vitro

**Figure 1:**
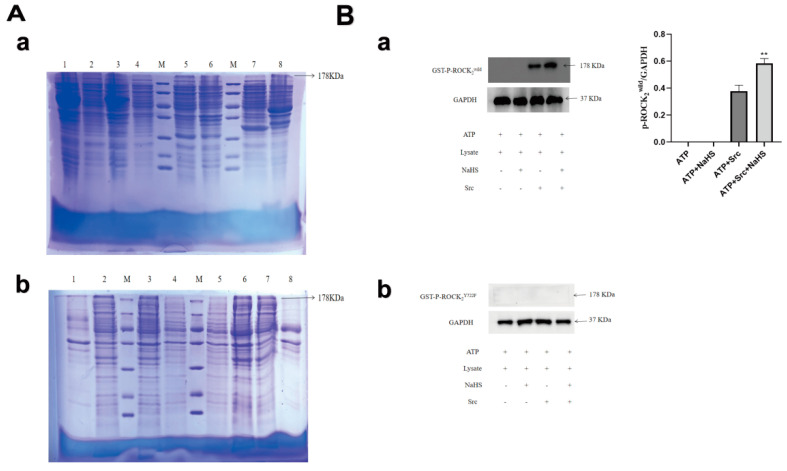
Expressions of GST-ROCK_2_^wild^ and GST-ROCK_2_^Y722F^ in *E. coli*, and effects of NaHS on their phosphorylation in vitro. (**A**) Coomassie brilliant blue staining assay. (**a**): M: marker, 1–2: induced lysate supernatant and precipitate of the empty *E. coli*, 3–4: induced lysate supernatant and precipitate of the empty plasmid transfected *E. coli*. 5–6: uninduced lysate supernatant and precipitate of the ROCK_2_^wild^-pGEX-6P-1-transfected *E. coli*, 7–8: uninduced lysate supernatant and precipitate of the ROCK_2_^Y722F^-pGEX-6P-1-transfected *E. coli.* (**b**): M: marker, 1–2: induced lysate precipitate and supernatant of the ROCK_2_^wild^-pGEX-6P-1-transfected *E. coli*, 3–4: induced lysate supernatant and precipitate of the ROCK_2_^Y722F^-pGEX-6P-1-transfected *E. coli,* 5–6: induced lysate precipitate and supernatant of the ROCK_2_^wild^-pGEX-6P-1-transfected *E. coli with* RIPA, 7–8: induced lysate precipitate and supernatant of the ROCK_2_^Y722F^-pGEX-6P -1-transfected *E. coli with* RIPA. (**B**) Effect of NaHS on phosphorylation of ROCK_2_ at Tyr722 in vitro (Western blot assay, mean ± SEM, *n* = 3). ATP: 100 μmol/L, Lys: 15 μL, NaHS: 100 μmol/L, Src: 100 ng. (**a**): in the lysate of the ROCK_2_^wild^-pGEX-6P-1-transfected *E. coli*; (**b**): in the lysate of the ROCK_2_^Y722F^-pGEX-6P-1- transfected *E. coli*. ** *p* < 0.01 vs. ATP + Src group.

As shown in Figure 1A(a), result of Coomassie brilliant blue staining showed that no target protein was detected in the lysate of empty *E. coli* and empty plasmid-transfected *E. coli*. Meanwhile, a significant different band appeared in the lysate precipitate of the ROCK_2_^wild^-pGEX-6P-1- or ROCK_2_^Y722F^-pGEX-6P-1-transfected *E. coli* induced by isopropyl-beta- D-thiogalactopyr-anoside (IPTG), and molecular weight of the band is about 178 KDa. The use of RIPA was not associated with an effect on the expression of the target protein, and glutathione S-transferase (GST)-ROCK_2_^wild^ or GST-ROCK_2_^Y722F^ was not detected in the lysate supernatant of the transfected *E. coli*. Our results showed that GST-ROCK_2_^wild^ and GST-ROCK_2_^Y722F^ were successfully expressed in *E. coli* transfected with prokaryotic plasmids. Perhaps, these two expressed proteins exist in the form of insoluble protein in *E. coli*, so they did not appear in the supernatants and cannot be further purified. Therefore, lysate of *E. coli* was used for phosphorylation experiment in vitro. To explore effect of H_2_S on phosphorylation of ROCK_2_ at Tyr722, lysate of the ROCK_2_^wild^-pGEX-6P-1- and the ROCK_2_^Y722F^-pGEX- 6P-1-transfected *E. coli* were used for phosphorylation in vitro. As shown in Figure 1B, Western blot assay showed that the tyrosine kinase, Src, induced significant phosphorylation of GST-ROCK_2_^wild^ (*p* < 0.01) in lysate of the ROCK_2_^wild^-pGEX-6P-1-transfected *E. coli*, and the NaHS, a H_2_S donor, markedly promoted the phosphorylation (*p* < 0.01), but NaHS was not observed to promote the phosphorylation of GST-ROCK_2_^Y722F^. The results indicated that H_2_S can promote the phosphorylation of GST-ROCK_2_ at Tyr722.

### 2.2. Effects of NaHS on Protein Expression and Activity of ROCK_2_ and Phosphorylation of ROCK_2_ at Tyr722 in RHNs

**Figure 2:**
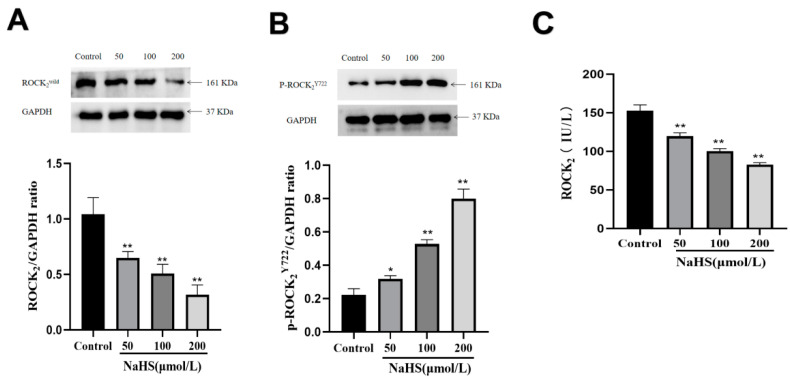
Effects of NaHS on ROCK_2_ expression and activity as well as phosphorylation of ROCK_2_ at Tyr722 in RHNs. (**A**) ROCK_2_ protein expression (Western blot assay, mean ± SEM, *n* = 3). (**B**) phosphorylation of ROCK_2_ at Tyr722 (Western blot assay, mean ± SEM, *n* = 3). (**C**) ROCK_2_ activity (ELISA, mean ± SEM, *n* = 3). * *p* < 0.05, ** *p* < 0.01 vs. control group.

As shown in Figure 2A,B, Western blot examination showed that NaHS (50, 100, 200 μmol/L) significantly inhibited the ROCK_2_ protein expression (*p* < 0.01, 0.01, 0.01) and promoted the phosphorylation of ROCK_2_ at Tyr722 (*p* < 0.05, 0.01, 0.01) in normal RHNs. Moreover, Figure 2C shows that NaHS markedly inhibited ROCK_2_ activity (*p* < 0.01, 0.01, 0.01). The results demonstrated that H_2_S not only inhibited the ROCK_2_ protein expression, but also promoted the phosphorylation of ROCK_2_ leading to the inhibition of ROCK_2_ activity.

### 2.3. NaHS-Induced Phosphorylation of ROCK_2_ or GFP-ROCK_2_ at Tyr722 in the Transfected RHNs

**Figure 3:**
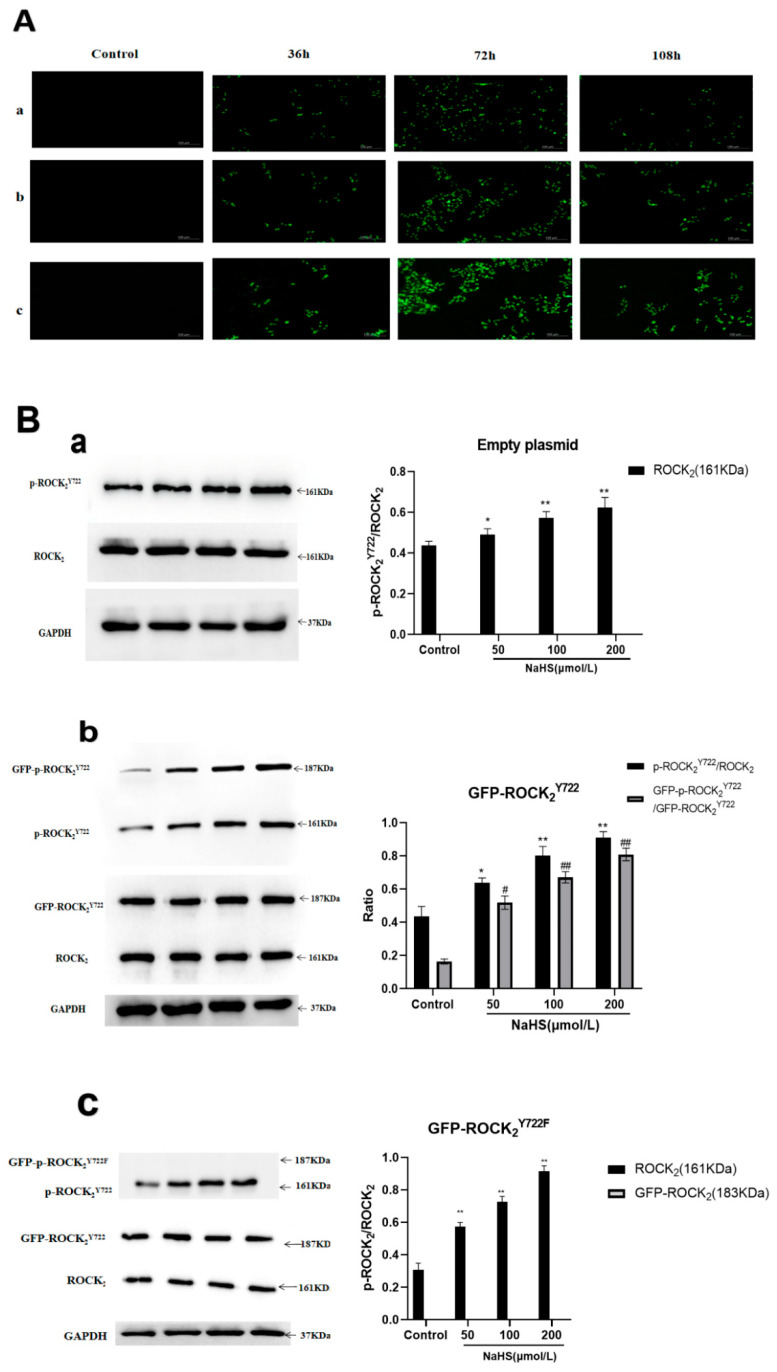
NaHS-induced phosphorylation of ROCK_2_ or GFP-ROCK_2_ at Tyr722 in the transfected RHNs. (**A**) Representative images of transfection. ((**a**): Empty plasmid-transfected RHNs; (**b**): ROCK_2_^Y722^-pEGFP-N1 plasmid-transfected RHNs; (**c**): ROCK_2_^Y722F^-pEGFP-N1 plasmid-transfected RHNs.) (GFP-tagged, 100 μm). (**B**) NaHS-promoted phosphorylation of ROCK_2_ at Tyr722 ((**a**): empty plasmid; (**b**): GFP-ROCK_2_^Y722^ group; (**c**): GFP-ROCK_2_^Y722F^ group) (Western blot assay, mean ± SEM, *n* = 3). * *p* < 0.05, ** *p* < 0.01 vs. control group (p-ROCK_2_^Y722^/ROCK_2_), # *p* < 0.05, ## *p* < 0.01 vs. control group (GFP-p-ROCK_2_^Y722^/GFP-ROCK_2_^Y722^).

Empty plasmid, ROCK_2_^Y722^-pEGFP-N1, and ROCK_2_^Y722F^-pEGFP-N1 were transfected into RHNs respectively. The expression of green fluorescent protein (GFP) was used to determine transfection efficiency. As shown in Figure 3A, transfection efficiency at 72 h was the highest than that at 36 h and 108 h. Therefore, transfection at 72 h was chosen for the following assays.

In the empty plasmid group, ROCK_2_ and p-ROCK_2_^Y722^ were detected, and NaHS (50, 100, 200 μmol/L) significantly increased the ratio of p-ROCK_2_^Y722^/ROCK_2_ (*p* < 0.05, 0.01, 0.01). In the GFP-ROCK_2_^Y722^ group, ROCK_2_, p-ROCK_2_^Y722^, GFP-ROCK_2_^Y722^, GFP-p-ROCK_2_^Y722^ were detected, and NaHS (50, 100, 200 μmol/L) markedly increased the ratios of both p-ROCK_2_^Y722^/ROCK_2_ and GFP-p-ROCK_2_^Y722^/GFP-ROCK_2_^Y722^ (*p* < 0.05, 0.01, 0.01). In the GFP-ROCK_2_^Y722F^ group, ROCK_2_, p-ROCK_2_^Y722^, GFP-ROCK_2_^Y722F^ were detected, NaHS (50, 100, 200 μmol/L) obviously increased the ratio of p-ROCK_2_^Y722^/ROCK_2_ (*p* < 0.05, 0.01, 0.01). The results demonstrated that H_2_S can promote phosphorylation of ROCK_2_ or GFP-ROCK_2_ at Tyr722 in RHNs.

### 2.4. Effects of NaHS on ROCK_2_ Activity in Eukaryotic Plasmid-Transfected RHNs

**Figure 4:**
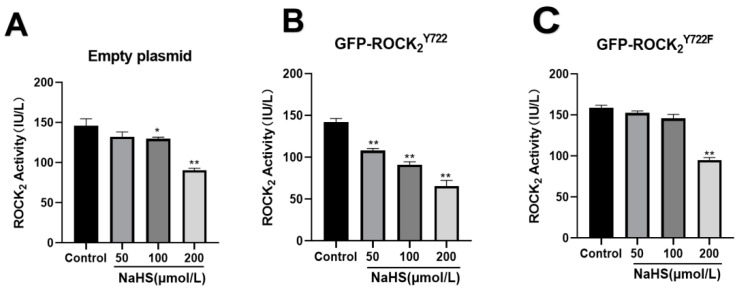
Effects of NaHS on the ROCK_2_ activities in the transfected RHNs with the empty plasmid, ROCK_2_^Y722^-pEGFP-N1 and ROCK_2_^Y722F^-pEGFP-N1, respectively (ELISA, mean ± SEM, *n* = 3). (**A**) Empty plasmid group. (**B**) GFP-ROCK_2_^Y722^ group. (**C**) GFP-ROCK_2_^Y722F^ group. * *p* < 0.05, ** *p* < 0.01 vs. control group.

As shown in Figure 4, in the empty plasmid group, NaHS (100, 200 μmol/L) had significant inhibitory effect on ROCK_2_ activity (*p* < 0.05, 0.01); in the GFP-ROCK_2_^Y722^ group, NaHS (50, 100, 200 μmol/L) obviously inhibited ROCK_2_ activity (*p* < 0.01, 0.01, 0.01); in the GFP-ROCK_2_^Y722F^ group, only 200 μmol/L NaHS inhibited ROCK_2_ activity (*p* < 0.05). The results suggested that ROCK_2_ Tyr722 may mediate in the H_2_S-inhibited ROCK_2_ activity in RHNs.

### 2.5. ROCK_2_ Tyr722 Mediated Protective Effect of NaHS on H/R Injury in Transfected RHNs

**Figure 5:**
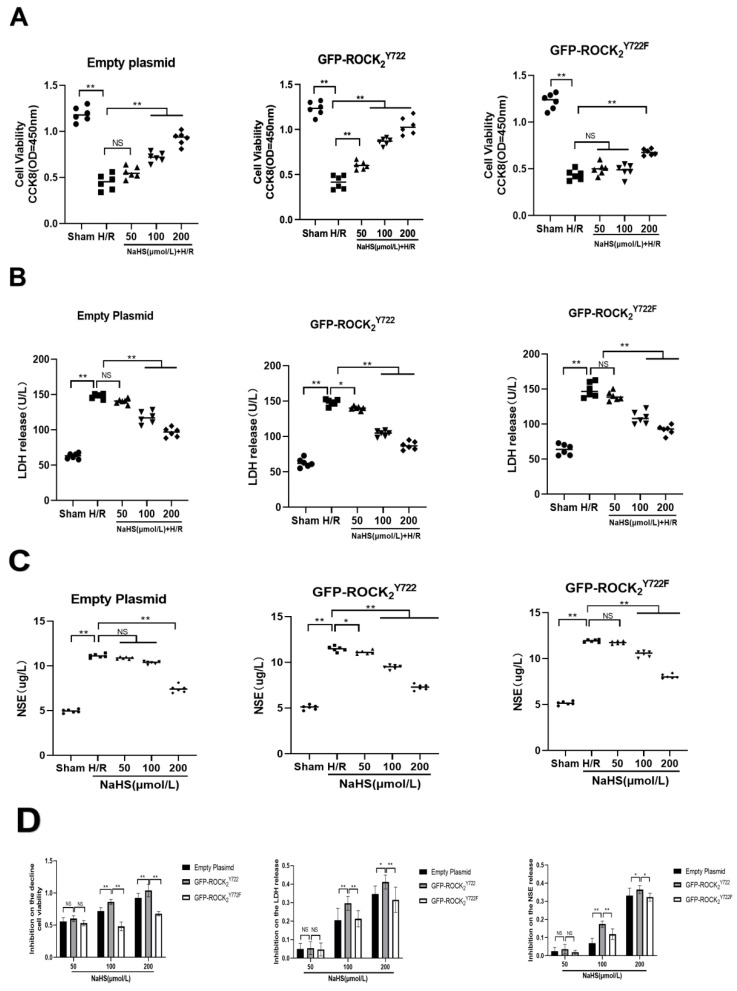
ROCK_2_ Tyr722-mediated protection of NaHS on H/R injury in transfected RHNs (mean ± SEM, *n* = 6). (**A**) Cell viability. (**B**) Release of LDH. (**C**) Release of NSE. * *p* < 0.05, ** *p* < 0.01 vs. H/R group. (**D**) Inhibitory effects on the decreased cell viability and released LDH and NSE. * *p* < 0.05, ** *p* < 0.01 vs. GFP-ROCK_2_^Y722^ group.

As shown in Figure 5, compared with Sham group, H/R injury significantly decreased cell viability (*p* < 0.01), and promoted the release of LDH (*p* < 0.01) and NSE (*p* < 0.01) in transfected RHNs. After treatment with NaHS (50, 100, 200 μmol/L), the protective effect against H/R injury was reflected in increased cell viability and inhibition of LDH and NSE release (Figure 5A–C). Compared with empty plasmid and GFP-ROCK_2_^Y722F^ groups, GFP-ROCK_2_^Y722^ group had a more significant effect on increasing cell viability and inhibiting LDH and NSE release (Figure 5D). Our results suggested that ROCK_2_ Tyr722 mediated protective effect of H_2_S against H/R injury in RHNs.

### 2.6. ROCK_2_ Tyr722 Mediated the NaHS-Increased Current of Big-Conductance Ca^2+^-Activated K^+^ Channel (BK_Ca_) in Transfected RHNs

**Figure 6:**
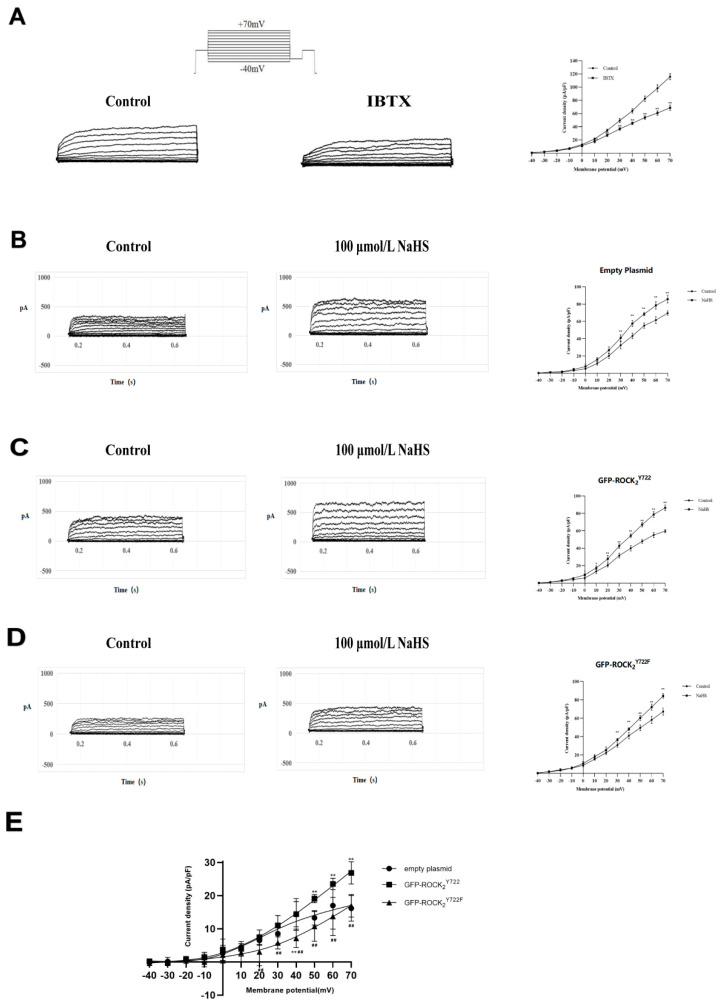
ROCK_2_ Tyr722 mediated NaHS-increased current of BK_Ca_ in transfected RHNs (whole−cell patch−clamp recording, mean ± SEM, *n* = 5). (**A**) The evoked outward current, and effect of IBTX. (**B**) Effect of NaHS on BK_Ca_ current in the empty plasmid group. (**C**) Effect of NaHS on BK_Ca_ current in the GFP−ROCK_2_^Y722^ group. (**D**) Effect of NaHS on BK_Ca_ current in the GFP−ROCK_2_^Y722F^ group. * *p* < 0.05, ** *p* < 0.01 vs. control group. (**E**) NaHS−increased BK_Ca_ currents in each plasmid−transfected RHNs. * *p* < 0.05, ** *p* < 0.01 vs. GFP−ROCK_2_^Y722^ group. ## *p* < 0.01 vs. empty plasmid group.

It is well-known that NaHS can open BK_Ca_ in various types of cells. In the present study, the whole-cell patch-clamp recording technique was used to record BK_Ca_ currents in RHNs. As shown in Figure 6A, a noise-like outward current was evoked in the RHNs by test pulses of −40 mV to +70 mV, and this outward current was voltage-dependent and significantly inhibited by 100 nmol/L iberiotoxin (IBTX), a BK_Ca_ specific blocker, suggesting that recorded current was a BK_Ca_ current. 

Figure 6B–D showed that NaHS (100 umol/L) significantly increased the BK_ca_ current in the transfected RHNs. However, NaHS increased BK_ca_ current in GFP-ROCK_2_^Y722^ group, which was obviously enhanced compared to empty plasmid and GFP-ROCK_2_^Y722F^ groups. This enhanced effect was significantly weakened in GFP-ROCK_2_^Y722F^ group (Figure 6E). The results suggested that ROCK_2_ Tyr722 mediated the H_2_S-increased current of BK_ca_ channel in RHNs.

## 3. Discussion

In the present study, it was found that H_2_S significantly promotes phosphorylation of ROCK_2_ at Tyr722, which mediates the protective effect of H_2_S against H/R injury in RHNs via inhibition of ROCK_2_ activity. Moreover, our study also demonstrated that Tyr722 of ROCK_2_ participates in the opening of the BK_Ca_ channel by H_2_S in RHNs, which may be of benefit to protect H/R injury in RHNs. 

The incidence of cerebrovascular disease is increasing year by year, and its disability rate and mortality rate are high, but the clinical treatment effect is not satisfactory [18,19]. As an important signaling pathway in cells, the RhoA-ROCK signaling pathway mediates many physiological and pathological processes of nerve cells, such as cell extension, contraction, and regeneration after injury [20,21,22]. Studies have shown that inhibition of the RhoA-ROCK signaling pathway can promote the growth of nerve cells, and ROCK_2_ plays a leading role [23]. Therefore, inhibition of the RhoA-ROCK signaling pathway plays a very important role in the treatment of stroke and has become a potential therapeutic target [24,25,26]. The growing body of evidence suggests that despite its past reputation as a noxious gas, H_2_S is rapidly emerging as a third gaseous transmitter, in addition to nitric oxide and carbon monoxide [27,28,29], and the physiological functions of H_2_S are receiving increasing attention. Previous experiments have shown that post-stroke exposure to H_2_S effectively lowered whole body temperature, prevented the upregulation of phagocytosis-specific protein annexin 1, and conferred neuroprotection in aged animals [30]. Although H_2_S has been shown to inhibit the RhoA-ROCK signaling pathway, the detailed mechanism remains unclear. Our recent study demonstrated that H_2_S promoted phosphorylation of RhoA at Ser188 to inhibit this pathway, but whether H_2_S can act on ROCK_2_ to inhibit this pathway has not been reported. 

In the present study, the prokaryotic plasmids, ROCK_2_^wild^-pGEX-6P-1 and ROCK_2_^Y722F^-pGEX-6P-1 were transfected into *E. coli*. The target proteins GST-ROCK_2_^wild^ and GST- ROCK_2_^Y722F^ were detected by Coomassie brilliant blue staining and Western blot assay. However, these two recombinant proteins were not detected in the lysate supernatant of the transfected *E. coli*, which may be due to the high molecular weight (consisting of more than 1300 amino acid residues). Therefore, the lysate of *E. coli* was used to carry out phosphorylation in vitro.

Phosphorylation of ROCK_2_ at Tyr722 occurs in the presence of kinase Src [31]. Our results showed that this phosphorylation happened in the absence of Src, indicating that ROCK_2_ did not undergo autophosphorylation. In the presence of Src, NaHS, a donor of H_2_S, significantly promoted phosphorylation of GST-ROCK_2_^wild^, but Src did not cause any phosphorylation of GST-ROCK_2_^Y722F^ in the presence or absence of Src or Src + NaHS. The results demonstrated that NaHS could promote phosphorylation of GST-ROCK_2_ at Tyr722. In the present study, it was further found that NaHS not only induced phosphorylation of ROCK_2_ at Tyr722 in RHNs, but also inhibited protein expression and activity of ROCK_2_. A previous study indicated that phosphorylation of ROCK_2_ at Tyr722 can reduce ROCK_2_ activity [32]. Thus, our results demonstrated that H_2_S can promote the phosphorylation of ROCK_2_ at Tyr722 to inhibit ROCK_2_ protein expression and activity in RHNs.

In the present study, by using lentivirus, eukaryotic recombinant ROCK_2_^Y722^-pEGFP -N1 and ROCK_2_^Y722F^-pEGFP-N1 plasmids as well as empty plasmid were transfected into the RHNs. Western blot assay indicated that NaHS significantly promoted the phosphorylation of ROCK_2_ and GFP-ROCK_2_^Y722^ but not GFP-ROCK_2_^Y722F^. Meanwhile, it was observed that inhibition of NaHS on ROCK_2_ activity was markedly enhanced in the GFP-ROCK_2_^Y722^ group compared to that in empty plasmid or the ROCK_2_^Y722F^ groups. These results indicated that ROCK_2_ Tyr722 mediates the H_2_S-inhibited ROCK_2_ activity in RHNs.

Phosphorylation of RhoA is involved in the protection of cerebral ischemic injury [33]. In the present study, it was found that transfection with the ROCK_2_^Y722^- pEGFP-N1 plasmid significantly increased the inhibitory effect of NaHS on the H/R injury-decreased cell viability and -released LDH and NSE in RHNs. Therefore, it could be concluded that phosphorylation of ROCK_2_ at Tyr722 also mediates protective effect of H_2_S against H/R injury in RHNs. It was well-known that inhibition of the RhoA-ROCK signaling pathway protects cerebral ischemia-reperfusion injury [34,35]. Combined with the above-mentioned that H_2_S promotes the phosphorylation of ROCK_2_ at Tyr722 to inhibit ROCK_2_ activity in RHNs, our results demonstrated that H_2_S protects RHNs from H/R injury through promoting phosphorylation of ROCK_2_ at Tyr722 to inhibit ROCK_2_ activity. 

Ca^2+^-activated K^+^ channels (K_Ca_) belong to the superfamily of potassium channels [36]. According to its conductance, K_ca_ is divided into three subseries: big conductance (BK_Ca_), intermediate-conductance (IK_Ca_), and small conductance (SK_Ca_) [37]. BK_Ca_ channels exist in a variety of non-excitatory and excitatory cells [38]. They are abundant in tissues and are important integrators in many biological functions that are essential for controlling the electrical activity of cells, hormone secretion, vascular regulation, auditory regulation of hair cells, or the generation of circadian rhythms [39]. Previous studies have shown that H_2_S can significantly increase BK_Ca_ current in neurons [40,41]. The protective effect of BK_Ca_ on vascular reactivity and calcium sensitivity may be mainly through the RhoA-ROCK pathway [42]. The present study showed that ROCK_2_ Tyr722 mediates the NaHS-increased current of BK_Ca_ channel in the RHNs. Together with a recent study that NaHS exerts neuroprotective effects via activating the BK_Ca_ [43], our results demonstrated that H_2_S promotes phosphorylation of ROCK_2_ at Tyr722 to open BK_Ca_ and protects the RHNs from H/R injury. 

The present study demonstrated that H_2_S regulated the phosphorylation of ROCK_2_ at Tyr722, and this regulation provided significant neuroprotection in rat neurons. There may probably be multiple mechanisms which are responsible for the neuroprotective effects of H_2_S. However, it is undeniable that the study in vitro cannot fully reflect the situation in vivo. The present study only conducted experiments in vitro, without any in vivo study, which is a limitation of our study. In addition, stroke is an aging-associated disease with comorbidities impacting seriously any drug therapy [44]; therefore, it should be more significant to research stroke in aged animal, which is another limitation of our study. Therefore, we will continue to explore the in vivo role of H_2_S in the phosphorylation of ROCK_2_ at Tyr722 resulting in protective effect against cerebral I/R injury, especially in aged animals in our future research. 

In conclusion, the present study provided evidence for the first time to demonstrate that H_2_S protects RHNs from H/R injury through promoting phosphorylation of ROCK_2_ at Tyr722 to inhibit ROCK_2_ activity and open BK_Ca_ channel. This finding may provide a basis for the use of H_2_S in the treatment of cerebrovascular diseases such as stroke.

## 4. Materials and Methods

### 4.1. Reagents

NaHS was obtained from Sigma Chemical (St. Louis, MO, USA); IPTG (catalogue number: G5042-5G), goat anti-rabbit IgG secondary antibody (catalogue number: G1213-100U), anti-GAPDH antibody (catalogue number: GB12002), marker (catalogue number: G2058-250UL), Coomassie blue R250 (catalogue number: GM1002), LDH assay kit (catalogue number: G1610-100T) were purchased from Servicebio (Wuhan, China); NSE assay kit (catalogue number: MM-0069R2) was obtained from Jiangsu Meimian Industrial, Co., Ltd. (Jiangsu, China); anti-ROCK_2_ antibody (catalogue number: ab125025), anti- ROCK_2_ (phospho Y722) antibody (catalogue number: ab182648), ATP (catalogue number: ab181719), recombinant Src (catalogue number: ab79635) were purchased from Abcam (San Francisco, CA, USA); 293T cell (catalogue number: c6008) was purchased from Beyotime Biotehchnology, Co. (Shanghai, China). 

### 4.2. Plasmids and Bacterium

The GST-tag ROCK_2_^wild^-pGEX-6p-1, ROCK_2_^Y722F^-pGEX-6p-1 prokaryotic recombin- ant plasmids and the GFP-tag ROCK_2_^Y722^-pEGFP-N1, ROCK_2_^Y722F^-pEGFP-N1 eukaryotic recombinant plasmids were obtained from Gene Create Biological Engineering, Co. (Wuhan, China) and constructed from rat genome. ROCK_2_^Y722F^, that is Tyr722 of ROCK_2_ was mutated to a phenylalanine (Phe or F) at the mRNA level. *E. coli* (BL21) was obtained from Gene Create Biological Engineering, Co. (Wuhan, China).

### 4.3. Expression of Prokaryotic Recombinant Protein GST-ROCK_2_^wild^ and GST-ROCK_2_^Y722F^

Prokaryotic recombinant plasmids ROCK_2_^wild^-pGEX-6P-1 and ROCK_2_^Y722F^-pGEX- 6P-1 were separately transformed into *E. coli*. Briefly, 20 µL of each plasmid was added to 100 µL of BL21 *E. coli* and placed on ice for 20 min. *E. coli* was kept at 42 °C for 90 s, 1 mL Luria-Bertani (LB) liquid medium was then added and the *E. coli* was cultured at 37 °C for 1 h. The *E. coli* was inoculated into LB solid medium and cultured at 37 °C for 24 h. The bacteria were inoculated into LB liquid medium. The medium was added with IPTG at a final concentration of 1 mM when the OD value was 0.8 and cultured at 37 °C for 6 h. The bacterial solution was collected and one part of the bacteria was sent to Shanghai Sangong Biological Co., Ltd. (Shanghai, China) for base sequencing, while the other part was crushed by ultrasonic crushing and RIPA, and total protein in the bacterial homogenate was determined using the BCA method. The homogenate was centrifugated at 10,000 rpm for 10 min. A total of 15 µL of supernatant or resuspended sediment specimen was separated by 8% SDS-PAGE at 120 mV for 40 min. The gel was stained with 200 mL Coomassie Brilliant Blue R-250 dye for 2 h. Then, discarding the staining solution, 200 mL decolorizing solution was added to decolorize the gel for 4 h until the protein band was clearly visible.

The sequencing results showed that the bases of site 722 of GST-ROCK_2_^wild^ in the ROCK_2_^wild^-pGEX-6P-1 plasmid-transfected *E. coli* were TAT, the bases of Tyr. The bases were mutated into TTT, the bases of phenylalanine (Phe, F), in the ROCK_2_^Y722F^-pGEX-6P-1 plasmid-transfected *E. coli*. 

### 4.4. In Vitro Phosphorylation Assay

About 15 μL protein specimen, 100 μmol/L ATP, and 100 ng Src kinase without or with 100 μmol/L NaHS were sequentially added to kinase buffer (20 mM Tris-Cl, 100 mM KCl, 2 mM EGTA, 5 mM MgCl_2_, pH 7.4), and then the mixture was shaken for 30 min at 37 °C. Then, SDS-PAGE loading buffer was added to terminate the phosphoryl transfer reaction. Phosphorylation of GST-ROCK_2_^wild^ or GST-ROCK_2_^Y722F^ proteins was examined by Western blotting assay.

### 4.5. Western Blotting

In each lane of the gels, 15 µg GST-ROCK_2_^wild^ and GST-ROCK_2_^Y722F^ were added, and electrophoresis was performed at 120 V for 1.5 h. After separation by 8% SDS–PAGE, the proteins were transferred to PVDF membranes. After blocking with 20% fetal bovine serum for 30 min, the membrane was washed with Tris-buffered saline with Tween (TBST) three times for 5 min each. The membranes were incubated overnight with anti-ROCK_2_ (phospho Y722) antibody at 4 °C and secondary antibody for 1 h at room temperature. ECL-Plus reagent was applied to the membrane, and chemiluminescence was visualized using a Fluor-S-max imager.

### 4.6. Determination of ROCK_2_ Activity

Mature RHNs were cultured for 6–8 days and the cells were oval or irregular in shape overall. RHNs were diluted with PBS at pH 7.2–7.4 to a concentration of 1 × 10^6^ cells /mL. The cells were crushed using an ultrasonic crusher with a power of 100 W, and the supernatant was collected after centrifugation of the crushed cells. The protein samples to be measured were added into 96-well plate and the blank wells and standard wells were set up, adjusting the blank wells to zero. The 96-well plate was incubated at 37 °C for 30 min. About 50 μL of termination solution was added to each well and then 50 μL of chromogenic agent A and 50 μL of chromogenic agent B were added. The mixture was shaken gently and mixed well, and allowed to develop for 15 min at 37 °C avoiding light. About 50 μL of termination solution was added to each well to terminate the reaction. The activity of ROCK_2_ was assayed at 450 nm on a microplate reader. The detection of activity should be completed within 15 min of the addition of the termination solution.

### 4.7. Lentivirus Transfection

The lentivirus vector was a second-generation vector that was cotransfected into 293T cells by empty, ROCK_2_^wild^-pEGFP-N1 and ROCK_2_^Y722F^-pEGFP-N1 plasmid, lentivirus packaging plasmid pCD/NLBH*DDD, and membrane protein expression plasmid PLTR-G. The RHNs were centrifuged at 10,000 r.p.m for 5 min, resuspended in 1 mL trypsin, and digested at 37 °C for 3 min. Cells were resuspended in 1 mL of fresh media and counted; 1 mL of this suspension (5 × 10^5^/mL cells) was added to each well in a 24-well plate. After adding 40 μL virus solution and 500 μL culture medium, the cells were cultured in an incubator. The medium was replaced with fresh culture medium after 24 h. Post-transfection, fluorescent protein expression was observed under an inverted fluorescence microscope at 36, 72, and 108 h to assess the infection efficiency.

### 4.8. Determination of Cell Viability

Cell viability was determined using a Cell Counting Kit-8 (CCK-8) cell viability assay kit. Mature RHNs cultured for 6–8 days were selected and their cell bodies were relatively full. Most of them are oval or irregular, and axons interweave with each other in a network. After H/R injury, 100 μL of RHNs suspension at a concentration of 2 × 10^4^ cells/well was added to the 96-well plates. The 96-well plates were incubated in an incubator for 24 h (37 °C, 5% CO_2_). A total of 10 μL of CCK-8 solution was added to each well and the 96-well plate was again incubated in an incubator for 24 h (37 °C, 5% CO_2_). The absorbance at 450 nm was measured using a microplate reader.

### 4.9. Determination of LDH

LDH was determined using an LDH Assay Kit. Mature RHNs cultured for 6–8 days were selected and their cell bodies were relatively full. After H/R injury, 100 μL of RHNs suspension at a concentration of 2 × 10^4^ cells/well was added to a 96-well plate. The original medium was removed, washed with PBS buffer, and low serum medium was added and incubated for 24 h. After incubation, the original medium was removed, washed again with PBS, and 120 μL of cell lysis working solution was added and incubated in an incubator for 60 min. The cell culture plate was centrifuged for 5 min, and 80 μL of lysis supernatant was added to a new 96-well plate and subsequently assayed. The absorbance of the supernatants was determined at 450 nm using a microplate reader. The results are expressed as U/L.

### 4.10. Determination of NSE

NSE was determined using a NSE Assay Kit. Mature RHNs cultured for 6–8 days were selected and their cell bodies were relatively full. After H/R injury, cells were centrifuged at 1000× *g* for 20 min and the supernatant was collected. Standard wells and sample wells were set up, and 50 μL of standard at different concentrations was added to each well, and 50 μL of sample to be tested was added to the sample wells. About 100 μL of horseradish peroxidase (HRP)-labeled detection antibody was added to each well, and the wells were sealed with sealing film and incubated in an incubator for 60 min. About 50 μL each of substrate A and B was added to each well, and incubated for 15 min at 37 °C, protecting from light. A total of 50 μL of termination solution was added to each well, and the OD value of each well was measured at 450 nm using a microplate reader. The results are expressed as μg/L.

### 4.11. Whole-Cell Patch-Clamp Technique

Recording electrodes were prepared from borosilicate glass pipettes and had a resistance of 5–9 MΩ. The recording electrodes were filled with recording solution. A 35 mm culture dish was prepared and the middle part was coated with an appropriate amount of petroleum jelly. The cultured RHNs crawls were removed, rinsed with PBS to remove dead cells and impurities, and placed in the middle of the culture dish coated with petroleum jelly. About 2 mL of extracellular fluid was added, and then the samples were placed on an inverted microscope. RHNs with smooth surface, large cytosol, and clear edges were found with a high-powered microscope and adjusted to the position so that they were in the center of the field of view. A tiny positive pressure was applied with a syringe before the glass microelectrode is placed in water. Adjust the micromanipulation to let the electrode drop slowly, use the computer software Clampex 10.5 to enter the membrane test window, click bath mode, and click the auto button in the multiClamp 700B window to maintain the entry baseline at about 0. The entry resistance of the electrode should be in the range of 4 to 8 MΩ. Gradually approach the electrode to the target cell first by micromanipulation under low magnification, and then move the electrode slowly toward the cell until contact is made. At this point, remove the positive pressure and apply a small negative pressure to the tip of the electrode, and the resistance will rise to about 1 GΩ if the cell is good. Click patch mode and then compensate the electrode capacitance with the amplifier. After the membrane was broken, the membrane capacitance and series resistance were compensated, and the whole-cell membrane capacitance current appeared, forming the whole-cell recording mode. The clamping voltage was set at −70 mV, I-V stimulation was given in voltage-clamp mode, and pulse stimulation was performed from −40 mV to 70 mV at a step of 10 mV, lasting approximately 150 ms at an interval of 3 s. NaHS was administered using a micropipette. The BK_Ca_ currents of RHNs were recorded on the recorder. Origin 8.0 software was used to plot concentration–respons curves. Standard extracellular fluid (NaCl 144 mM, KCl 6 mM, MgCl_2_·6H_2_O 1.2 mM, CaCl_2_ 2 mM, HEPES 10 mM, D-glucose 10 mM and 4-AP 5 mM) was adjusted with NaOH to pH 7.4, standard intracellular fluid (K-glutamine 110 mM, KCl 20 mM, MgCl_2_·6H_2_O 3 mM, EGTA 0.1 mM, Na_2_ATP 3 mM, HEPES 10 mM and D-glucose10 mM) was adjusted with KOH to pH 7.0.

### 4.12. H/R Injury

After the cells were placed in a conventional incubator at 37 °C for 2 h, the medium of the cells was changed to sugar-free medium and placed in a hypoxic incubator (37 °C, 1% O_2_, 5% CO_2_) for 8 h. At the end of the incubation, the sugar-free medium was discarded and replaced with complete medium and placed in a normal incubator for 6 h for the next experiment.

### 4.13. Statistical Analysis

The data are expressed as mean ± SEM. one-way analysis of variance (ANOVA), or two-way ANOVA was used to conduct data analysis. *p* < 0.05 was considered as significant difference.

## Data Availability

The data presented in this study are available on request from the corresponding author.

## References

[1] Mandalaneni K., Rayi A., Jillella D.V. (2022). Stroke Reperfusion Injury. StatPearls.

[2] Hussein A.M., Khaled H.K., Seisa M.O., Baiomy A., Mohamed M.A., Eltantawy D., Mahmoud A.A., Sheashaa H.A., Sobh M.A. (2014). Possible role of nitric oxide in hepatic injury secondary to renal ischemia-reperfusion (I/R) injury. Gen. Physiol. Biophys..

[3] Jiang S., Dandu C., Geng X. (2020). Clinical application of nitric oxide in ischemia and reperfusion injury: A literature review. Brain Circ..

[4] Guan X., Li X., Yang X., Yan J., Shi P., Ba L., Cao Y., Wang P. (2019). The neuroprotective effects of carvacrol on ischemia/reperfusion-induced hippocampal neuronal impairment by ferroptosis mitigation. Life Sci..

[5] Wen Y.D., Wang H., Zhu Y.Z. (2018). The Drug Developments of Hydrogen Sulfide on Cardiovascular Disease. Oxid. Med. Cell. Longev..

[6] Cao X., Cao L., Ding L., Bian J.S. (2018). A New Hope for a Devastating Disease: Hydrogen Sulfide in Parkinson’s Disease. Mol. Neurobiol..

[7] Zhang Y., Li K., Wang X., Ding Y., Ren Z., Fang J., Sun T., Guo Y., Chen Z., Wen J. (2021). CSE-Derived H2S Inhibits Reactive Astrocytes Proliferation and Promotes Neural Functional Recovery after Cerebral Ischemia/Reperfusion Injury in Mice Via Inhibition of RhoA/ROCK2 Pathway. ACS Chem. Neurosci..

[8] Han J., Chen Z.W., He G.W. (2013). Acetylcholine- and sodium hydrosulfide-induced endothelium-dependent relaxation and hyperpolarization in cerebral vessels of global cerebral ischemia-reperfusion rat. J. Pharmacol. Sci..

[9] Wang M., Hu Y., Fan Y., Guo Y., Chen F., Chen S., Li Q., Chen Z. (2016). Involvement of Hydrogen Sulfide in Endothelium-Derived Relaxing Factor-Mediated Responses in Rat Cerebral Arteries. J. Vasc. Res..

[10] Wu D., Wang J., Li H., Xue M., Ji A., Li Y. (2015). Role of Hydrogen Sulfide in Ischemia-Reperfusion Injury. Oxid. Med. Cell. Longev..

[11] Lai A.Y., McLaurin J. (2018). Rho-associated protein kinases as therapeutic targets for both vascular and parenchymal pathologies in Alzheimer’s disease. J. Neurochem..

[12] Roloff F., Scheiblich H., Dewitz C., Dempewolf S., Stern M., Bicker G. (2015). Enhanced neurite outgrowth of human model (NT2) neurons by small-molecule inhibitors of Rho/ROCK signaling. PLoS ONE.

[13] Scheiblich H., Bicker G. (2017). Regulation of Microglial Phagocytosis by RhoA/ROCK-Inhibiting Drugs. Cell. Mol. Neurobiol..

[14] Wen J.Y., Gao S.S., Chen F.L., Chen S., Wang M., Chen Z.W. (2019). Role of CSE-Produced H2S on Cerebrovascular Relaxation via RhoA-ROCK Inhibition and Cerebral Ischemia-Reperfusion Injury in Mice. ACS Chem. Neurosci..

[15] Sawada N., Itoh H., Yamashita J., Doi K., Inoue M., Masatsugu K., Fukunaga Y., Sakaguchi S., Sone M., Yamahara K. (2001). cGMP-dependent protein kinase phosphorylates and inactivates RhoA. Biochem. Biophys. Res. Commun..

[16] Chen Y., Wen J., Chen Z. (2021). H2S protects hippocampal neurons against hypoxia-reoxygenation injury by promoting RhoA phosphorylation at Ser188. Cell Death Discov..

[17] Lee H.H., Tien S.C., Jou T.S., Chang Y.C., Jhong J.G., Chang Z.F. (2010). Src-dependent phosphorylation of ROCK participates in regulation of focal adhesion dynamics. J. Cell Sci..

[18] Onyike C.U. (2006). Cerebrovascular disease and dementia. Int. Rev. Psychiatry.

[19] Narasimhan M., Schwartz R., Halliday G. (2022). Parkinsonism and cerebrovascular disease. J. Neurol. Sci..

[20] Chen S., Wang H., Xu H., Zhang Y., Sun H. (2020). Electroacupuncture promotes axonal regrowth by attenuating the myelin-associated inhibitors-induced RhoA/ROCK pathway in cerebral ischemia/reperfusion rats. Brain Res..

[21] Fujita Y., Yamashita T. (2014). Axon growth inhibition by RhoA/ROCK in the central nervous system. Front. Neurosci..

[22] Iyer M., Subramaniam M.D., Venkatesan D., Cho S.G., Ryding M., Meyer M., Vellingiri B. (2021). Role of RhoA-ROCK signaling in Parkinson’s disease. Eur. J. Pharmacol..

[23] Koch J.C., Tonges L., Barski E., Michel U., Bahr M., Lingor P. (2014). ROCK2 is a major regulator of axonal degeneration, neuronal death and axonal regeneration in the CNS. Cell Death Dis..

[24] Sladojevic N., Yu B., Liao J.K. (2017). ROCK as a therapeutic target for ischemic stroke. Expert Rev. Neurother..

[25] Lu W., Chen Z., Wen J. (2021). RhoA/ROCK signaling pathway and astrocytes in ischemic stroke. Metab. Brain Dis..

[26] Lu E., Wang Q., Li S., Chen C., Wu W., Xu Y.X.Z., Zhou P., Tu W., Lou X., Rao G. (2020). Profilin 1 knockdown prevents ischemic brain damage by promoting M2 microglial polarization associated with the RhoA/ROCK pathway. J. Neurosci. Res..

[27] Sycheva M., Sustarich J., Zhang Y., Selvaraju V., Geetha T., Gearing M., Babu J.R. (2019). Pro-Nerve Growth Factor Induces Activation of RhoA Kinase and Neuronal Cell Death. Brain Sci..

[28] Murphy B., Bhattacharya R., Mukherjee P. (2019). Hydrogen sulfide signaling in mitochondria and disease. FASEB J..

[29] Kanagy N.L., Szabo C., Papapetropoulos A. (2017). Vascular biology of hydrogen sulfide. Am. J. Physiol. Cell Physiol..

[30] Joseph C., Buga A.M., Vintilescu R., Balseanu A.T., Moldovan M., Junker H., Walker L., Lotze M., Popa-Wagner A. (2012). Prolonged gaseous hypothermia prevents the upregulation of phagocytosis-specific protein annexin 1 and causes low-amplitude EEG activity in the aged rat brain after cerebral ischemia. J. Cereb. Blood Flow Metab..

[31] Lee H.H., Chang Z.F. (2008). Regulation of RhoA-dependent ROCKII activation by Shp2. J. Cell Biol..

[32] Kim H.J., Kim M.J., Mostafa M.N., Park J.H., Choi H.S., Kim Y.S., Choi E.K. (2020). RhoA/ROCK Regulates Prion Pathogenesis by Controlling Connexin 43 Activity. Int. J. Mol. Sci..

[33] Loirand G., Guilluy C., Pacaud P. (2006). Regulation of Rho proteins by phosphorylation in the cardiovascular system. Trends Cardiovasc. Med..

[34] Xiao J., Zhu X., Kang B., Xu J., Wu L., Hong J., Zhang Y., Ni X., Wang Z. (2015). Hydrogen Sulfide Attenuates Myocardial Hypoxia-Reoxygenation Injury by Inhibiting Autophagy via mTOR Activation. Cell Physiol. Biochem..

[35] Guo Y., Yu X.M., Chen S., Wen J.Y., Chen Z.W. (2020). Total flavones of Rhododendron simsii Planch flower protect rat hippocampal neuron from hypoxia-reoxygenation injury via activation of BKCa channel. J. Pharm. Pharmacol..

[36] Gueguinou M., Chantome A., Fromont G., Bougnoux P., Vandier C., Potier-Cartereau M. (2014). KCa and Ca(2+) channels: The complex thought. Biochim. Biophys. Acta.

[37] Dong D.L., Bai Y.L., Cai B.Z. (2016). Calcium-Activated Potassium Channels: Potential Target for Cardiovascular Diseases. Adv. Protein Chem. Struct. Biol..

[38] Zhang I., Hu H. (2020). Store-Operated Calcium Channels in Physiological and Pathological States of the Nervous System. Front. Cell Neurosci..

[39] Choi S., Kim J.A., Li H.Y., Shin K.O., Oh G.T., Lee Y.M., Oh S., Pewzner-Jung Y., Futerman A.H., Suh S.H. (2016). KCa 3.1 upregulation preserves endothelium-dependent vasorelaxation during aging and oxidative stress. Aging Cell.

[40] Sitdikova G.F., Weiger T.M., Hermann A. (2010). Hydrogen sulfide increases calcium-activated potassium (BK) channel activity of rat pituitary tumor cells. Pflugers Arch..

[41] Sitdikova G.F., Fuchs R., Kainz V., Weiger T.M., Hermann A. (2014). Phosphorylation of BK channels modulates the sensitivity to hydrogen sulfide (H2S). Front. Physiol..

[42] Wu Y., Yue Z., Wang Q., Lv Q., Liu H., Bai Y., Li S., Xie M., Bao J., Ma J. (2019). BKCa compensates impaired coronary vasoreactivity through RhoA/ROCK pathway in hind-limb unweighted rats. FASEB J..

[43] Wen J.Y., Zhang J., Chen S., Chen Y., Zhang Y., Ma Z.Y., Zhang F., Xie W.M., Fan Y.F., Duan J.S. (2021). Endothelium-derived hydrogen sulfide acts as a hyperpolarizing factor and exerts neuroprotective effects via activation of large-conductance Ca(2+) -activated K(+) channels. Br. J. Pharmacol..

[44] Popa-Wagner A., Dumitrascu D.I., Capitanescu B., Petcu E.B., Surugiu R., Fang W.H., Dumbrava D.A. (2020). Dietary habits, lifestyle factors and neurodegenerative diseases. Neural. Regen Res..

